# Supplementary material for: MetaRibo-Seq measures translation in microbiomes
Source: Nat Commun. 2020 Jun 29;11:3268. doi: 10.1038/s41467-020-17081-z (PMC7324362; doi:10.1038/s41467-020-17081-z)
Supplement: Supplementary file 10 — Supplementary Data 7 [file 41467_2020_17081_MOESM10_ESM.zip › File2/Confidence_High_Taxonomy/57030_out.krona.html]

Javascript must be enabled to view this page.

members
magnitude
magnitudeUnassigned
count
unassigned
taxon
rank

57030\_out

4

1

SRS013687\_contig\_number\_contig-100\_11482.102382

2
3
superkingdom

3
phylum
1239

class
3
186801

186802
order
3

3
family
186803

species
3
1952162

SRS011084\_contig\_number\_14212SRS058070\_contig\_number\_20910SRS147377\_contig\_number\_76
